# Supplementary material for: Molecular Epidemiology and Evolution of Coxsackievirus A9
Source: Viruses. 2022 Apr 15;14(4):822. doi: 10.3390/v14040822 (PMC9024771; doi:10.3390/v14040822)
Supplement: Supplementary file 1 [file viruses-14-00822-s001.zip › viruses-1645085-supl. tables/viruses-1645085-Table S1.pdf]

Table S1. The primers designed in this study for amplifying and sequencing the whole genome sequence of CVA9

Name	Location			Direction	Source
0001S48	-	GGGGACAAGTTTGTACAAAAAAGCAG	0–700bp	Forward	-
CVA9-541A	523–541	CTGCAGAGTTGCCCGTTAC		Reverse	This study
CVA9-520S	520–538	GTCGTAACGGGCAACTCTG	600–1500bp	Forward	This study
CVA9-1205A	1186–1205	CTGGGAACTTCCACCACCAC		Reverse	This study
CVA9-1185S	1185–1206	GGTGGTGGAAGTTCCCAGA	1300–2200bp	Forward	This study
CVA9-1562A	1543–1562	GCATCACTATGGTGGCACTG		Reverse	This study
CVA9-1543S	1543–1562	CAGTGCCACCATAGTGATGC	2000–2800bp	Forward	This study
CVA9-2343A	2323–2343	GGTGGGACAATCATACCAGTC		Reverse	This study
CVA9-2407S	2407–2426	GATGTTAAGAGACACTCCAT	2700–3600bp	Forward	This study
CVA9-2839A	2820–2839	ATCACAAGTCGGCAAGATCC		Reverse	This study
CVA9-3256S	3256–3275	CAACACCCATCACAGACACC	3350–4200bp	Forward	This study
CVA9-3652A	3629–3652	GAACCTCCCCATGGTTACCT		Reverse	This study
CVA9-3829S	3829–3848	ACAACTTGGGAACGCTTTTG	4000–4900bp	Forward	This study
CVA9-4841A	4822–4841	GCAAGGAGGTTTCACTTCGA		Reverse	This study
CVA9-3963S	3963–3992	GCATTGGTGATTGTGGTGAG	4800–5700bp	Forward	This study
CVA9-5821A	5802–5821	ATAAACCTGGGTGGTACCCC		Reverse	This study
CVA9-5624S	5624–5643	GATGGCACCAATTTGGAGTT	5600–6300bp	Forward	This study
CVA9-6138A	6119–6138	TACTCCCCTCAAAGACGTGG		Reverse	This study
CVA9-5943S	5943–5962	GCAGCCCTCCTCAAACACTA	5900–6800bp	Forward	This study
CVA9-6837A	6818–6837	TACTCCCCTCAAAGACGTGG		Reverse	This study
CVA9-7054S	7054–7073	AGACAAAGGGGAGTGCTTCA	6500–7500bp	Forward	This study
7500A	-	GGGGACCACTTTGTACAAGAAAGCTG		Reverse	-
