# Supplementary material for: Molecular Epidemiology and Evolution of Coxsackievirus A9
Source: Viruses. 2022 Apr 15;14(4):822. doi: 10.3390/v14040822 (PMC9024771; doi:10.3390/v14040822)
Supplement: Supplementary file 1 [file viruses-14-00822-s001.zip › viruses-1645085-supl. tables/viruses-1645085-Table S2.pdf]

Table S2. 128 isolates used for phylogenetic analysis of CVA9

No.	genotype	Sample ID	Isolation Year	Country of Origin	Sample Type	Clinical Symptoms	GenBank Accession	Source
1	A	Griggs	1950	USA	Clone	N/A	D00627	GenBank
2	B	CO62	1962	UK	Unknown	N/A	JN996502	GenBank
3	C	CO79	1979	UK	Unknown	N/A	JN996501	GenBank
4	D	Cuba47of93	1993	Cuba	CSF	Neuropathy	AY466031	GenBank
5	D	Cuba23of00	2000	Cuba	CSF	Meningitis	AY466033	GenBank
6	D	Cuba689of93	1993	Cuba	CSF	Neuropathy	AY466032	GenBank
7	D	Cuba35of93	1993	Cuba	CSF	Neuropathy	AY466030	GenBank
8	D	Cuba270of90	1990	Cuba	CSF	Meningitis	AY466024	GenBank
9	D	Cuba267of90	1990	Cuba	CSF	Meningitis	AY466029	GenBank
10	D	Cuba163of91	1991	Cuba	CSF	Meningitis	AY466028	GenBank
11	D	Cuba135of91	1991	Cuba	CSF	Meningitis	AY466027	GenBank
12	D	Cuba96of91	1991	Cuba	CSF	Meningitis	AY466022	GenBank
13	D	Cuba45of91	1991	Cuba	CSF	Meningitis	AY466025	GenBank
14	D	Cuba450of90	1990	Cuba	CSF	Meningitis	AY466023	GenBank
15	D	Cuba100of91	1991	Cuba	CSF	Meningitis	AY466026	GenBank
16	D	2-MRS2013	2013	France	CSF	AM	KM201659	GenBank
17	D	RO-609-4-80	1980	Romania	stool	N/A	LS451285	GenBank
18	E	CO85	1985	UK	CSF	N/A	JN996500	GenBank
19	F	CO87	1987	UK	Unknown	N/A	JN996499	GenBank
20	F	A16-52PD	2010	India	Stool	Persistent diarrhea	KF177114	GenBank
21	F	NIV56210	2005	India	Stool	AFP	KF412927	GenBank
22	F	A450D	2011	India	Unknown	Acute diarrhea	JX513563	GenBank
23	F	263/CSF/CVA9/RUS/Omsk/2012	2012	Russia	CSF	AM	KU133575	GenBank
24	F	382/CSF/CVA9/RUS/Omsk/2013	2013	Russia	CSF	AM	KU133620	GenBank
25	G	06.109.3344	2006	Australia	Unknown	AM	FJ868282	GenBank
26	G	NSW-V20-2008-CVA9	2008	Australia	Stool	N/A	MF678309	GenBank
27	G	NSW-V56-2006-CVA9	2006	Australia	Stool	N/A	MF678346	GenBank
28	G	CVA9/EBMSV0003/THA/2010	2010	Thailand	Throat	Respiratory diseases	KU574638	GenBank
29	G	Patient FJ98-90	2004	FJ/China	Unknown	Unknown	AY573577	GenBank
30	G	Patient FJ00-127	2004	FJ/China	Unknown	Unknown	AY573578	GenBank
31	G	97089/SD/CHN/1997/CA9	1997	SD/China	Unknown	N/A	GQ329727	GenBank
32	G	01332/SD/CHN/2001/CA9	2001	SD/China	Unknown	N/A	GQ329730	GenBank
33	G	CVA9_CF027040_FRA07	2007	France	Throat	N/A	HF948081	GenBank
34	G	064/LY/CHN/AM/10/CA9	2010	SD/China	CSF	AM	KF150145	GenBank
35	G	CVA9/FJZZ122/CHN/2011	2011	FJ/China	Stool	HFMD	MG922505	GenBank
36	G	YZ047/SD/CHN/2005/CA9	2005	SD/China	Stool	HFMD	GQ246517	GenBank
37	G	CVA9_Alberta_2013	2013	Canada	CSF	AM	JQ837913	GenBank
38	G	97186/SD/CHN/1997/CA9	1997	SD/China	Stool	HFMD	GQ329728	GenBank
39	G	00365/SD/CHN/2000/CA9	2000	SD/China	Stool	HFMD	GQ329729	GenBank
40	G	04318/SD/CHN/2004/CA9	2004	SD/China	Stool	HFMD	GQ329731	GenBank
41	G	USA/MI/2005-23029	2005	USA	Unknown	N/A	MH752987	GenBank
42	G	NSW-V14-2009-CVA9	2009	Australia	Stool	N/A	MF678303	GenBank

43	G	1-D7-CA9	2008	TW/China	Throat	Rash	KT353721	GenBank
44	G	61238-7074	2008	TW/China	Throat	Rash	MF422557	GenBank
45	G	JB14080245	2008	GD/China	Stool	HFMD	KC867074	GenBank
46	G	Gansu05-1/GS/CHN/2005	2005	GS/China	CSF	AM	GQ294574	GenBank
47	G	A242/YN/CHN/2009	2009	YN/China	Feces	AM	KM890278	GenBank
48	G	2249/PL12/3013	2013	Poland	Stool	AFP	KU189253	GenBank
49	G	CVA9/PMKA0512/THA/2010	2010	Thailand	Throat	Respiratory diseases	KU574637	GenBank
50	G	CVA9/PMKA0420/THA/2010	2010	Thailand	Throat	Respiratory diseases	KU574636	GenBank
51	G	CVA9_Alberta_2010	2010	Canada	CSF	AM	JQ837914	GenBank
52	G	253/JN/CHN/AM/10/CA9	2010	SD/China	CSF	AM	KF246748	GenBank
53	G	A14/YN/CHN/2010	2010	YN/China	CSF	AM	MF098518	GenBank
54	G	A73/YN/CHN/2010	2010	YN/China	CSF	AM	MF098523	GenBank
55	G	NSW-V41-2013-CVA9	2013	Australia	Stool	N/A	MF678330	GenBank
56	G	P73/ZS/CHN/2011	2011	China/GX	Stool	HFMD	MN018193	GenBank
57	G	024/LS/CHN/AM/08/CA9	2008	SD/China	CSF	AM	KF246747	GenBank
58	G	USA/2019-23312	2019	USA	Liver	Unknown	MN896915	GenBank
59	G	USA/2019-23313	2019	USA	Liver	Unknown	MN896916	GenBank
60	G	USA/2019-23314	2019	USA	Liver	Unknown	MN896917	GenBank
61	G	CLI-B1-10-CV-A9	2017	UK	CSF	AM	MT641360	GenBank
62	G	16C2	2016	USA	Unknown	N/A	KY674974	GenBank
63	G	16C8	2016	USA	Unknown	N/A	KY674976	GenBank
64	G	CVA9/FJQZ126/CHN/2011	2011	FJ/China	Stool	HFMD	MG922506	GenBank
65	G	A108/YN/CHN/2009	2009	YN/China	Feces	AM	KM890277	GenBank
66	G	A744/YN/CHN/2009	2009	YN/China	CSF	AM	MN686207	GenBank
67	G	CVA9/FJZZ056/CHN/2011	2011	FJ/China	Stool	HFMD	MG922504	GenBank
68	G	CV-A9/P351/2013/China	2013	ZJ/China	CSF	AM	KP289437	GenBank
69	G	LHY03	2010	JS/China	Unknown	HFMD	KP266574	GenBank
70	G	JB141230009	2012	GD/China	Stool	HFMD	KC867075	GenBank
71	G	071/LY/CHN/AM/10/CVA9	2010	SD/China	CSF	AM	KF150146	GenBank
72	G	C1/LC/2019	2019	SD/China	CSF	AM	MT950542	GenBank
73	G	C23/LC/CHN/2019	2019	SD/China	CSF	AM	MT950564	GenBank
74	G	C39/LC/CHN/2018	2018	SD/China	CSF	AM	MT646125	GenBank
75	G	ZG2018-176-CVA9	2018	SC/China	Stool	HFMD	MW179455	GenBank
76	G	JB141230166	2012	GD/China	Stool	HFMD	KC867076	GenBank
77	G	JB141230172	2012	GD/China	Stool	HFMD	KC867077	GenBank
78	G	JB141230177	2012	GD/China	Stool	HFMD	KC867078	GenBank
79	G	CVA9/P56/2013/China	2013	ZJ/China	Stool	HFMD	KP289434	GenBank
80	G	CVA9/FJZ463/CHN/2020	2014	FJ/China	Stool	HFMD	MG922507	GenBank
81	G	DY2016-50-CVA9	2014/2019	SC/China	Stool	N/A	MW179442	GenBank
82	G	CV-A9/P220/2013/China	2013	ZJ/China	Stool	HFMD	KP290111	GenBank
83	G	A34-YN-CHN-2014	2014	YN/China	Stool	HFMD	LC013414	GenBank
84	G	A34-YN-CHN-2014	2014	YN/China	Stool	HFMD	LC411969	GenBank
85	G	120-YN-CHN-2014JK	2014	YN/China	Homo	Healthy children	LC120871	GenBank
86	G	CVA9/FJQZ182/CHN/2012	2012	FJ/China	Stool	HFMD	MG922503	GenBank
87	G	C28/LC/CHN/2019	2019	SD/China	CSF	AM	MT950569	GenBank
88	G	C38/LC/CHN/2019	2019	SD/China	CSF	AM	MT950579	GenBank

89	G	C18-YN-CHN-2018	2018	YN/China	Stool	HFMD	LC481444	GenBank
90	G	C20-YN-CHN-2018	2018	YN/China	Stool	HFMD	LC481445	GenBank
91	G	DY2016-18-CVA9	2016	SC/China	Homo	Healthy children	MW179429	GenBank
92	G	DY2016-21-CVA9	2014/2019	SC/China	Unknown	Healthy child	MW179431	GenBank
93	G	A280-KM-CHN-CVA9	2018	YN/China	Unknown	HFMD	LC516768	GenBank
94	G	C4/LC/CHN/2019	2019	SD/China	CSF	AM	MT950545	GenBank
95	G	C16/LC/CHN/2019	2019	SD/China	CSF	AM	MT950557	GenBank
96	G	SF149	2016	SC/China	Stool	Healthy children	LC361281	GenBank
97	G	ZJ154	2016	ZJ/China	Stool	N/A	LC361282	GenBank
98	G	ZJ160	2016	ZJ/China	Stool	N/A	LC361283	GenBank
99	G	China/GS/2015/05	2015	GS/China	Stool	HFMD	OM885385	This study
100	G	China/GS/2015/61	2015	GS/China	Stool	HFMD	OM885386	This study
101	G	China/GS/2015/254	2015	GS/China	Stool	HFMD	OM885387	This study
102	G	China/GX/2019/175	2019	GX/China	Stool	HFMD	OM885388	This study
103	G	China/HeB/2019/98	2019	HeB/China	Stool	HFMD	OM885389	This study
104	G	China/SaX/2019/49	2019	SaX/China	Stool	HFMD	OM885390	This study
105	G	China/SD/2015/41	2015	SD/China	Stool	HFMD	OM885392	This study
106	G	China/SD/2016/246	2016	SD/China	Stool	HFMD	OM885391	This study
107	G	China/SD/2019/074	2019	SD/China	Stool	HFMD	OM885393	This study
108	G	China/SD/2019/114	2019	SD/China	Stool	HFMD	OM885394	This study
109	G	China/SD/2019/130	2019	SD/China	Stool	HFMD	OM885395	This study
110	G	China/SD/2019/323	2019	SD/China	Stool	HFMD	OM885396	This study
111	G	China/SD/2019/403	2019	SD/China	Stool	HFMD	OM885397	This study
112	G	China/SD/2019/437	2019	SD/China	Stool	HFMD	OM885398	This study
113	G	China/XJ/2015/83	2015	XJ/China	Stool	HFMD	OM885399	This study
114	G	China/XJ/2017/117	2015	XJ/China	Stool	HFMD	OM885400	This study
115	G	China/XJ/2018/101	2015	XJ/China	Stool	HFMD	OM885402	This study
116	G	China/YN/2014/131	2014	YN/China	Stool	HFMD	OM885403	This study
117	H	99.358.0740	1999	Australia	Unknown	N/A	GU142874	GenBank
118	I	CF315290013_FRA15-10-16_CV-A9	2015	France	CSF	meningitis	MK086198	GenBank
119	I	408/CSF/CVA9/RUS/Omsk/2013	2013	Russia	CSF	AM	KU133635	GenBank
120	I	SPb_5005/13EVI/RKa-32/14/RU	2013	Russia	CSF	AM	KU841453	GenBank
121	I	297/CSF/CVA9/RUS/Omsk/2012	2012	Russia	CSF	AM	KU133591	GenBank
122	I	302/CSF/CVA9/RUS/Omsk/2012	2012	Russia	CSF	AM	KU133594	GenBank
123	I	299/CSF/CVA9/RUS/Omsk/2012	2012	Russia	CSF	AM	KU133593	GenBank
124	I	309/CSF/CVA9/RUS/Omsk/2012	2012	Russia	CSF	AM	KU133597	GenBank
125	I	270/CSF/CVA9/RUS/Omsk/2012	2012	Russia	CSF	AM	KU133580	GenBank
126	I	325/CSF/CVA9/RUS/Omsk/2012	2012	Russia	CSF	AM	KU133606	GenBank
127	I	China/XJ/2018/99	2018	XJ/China	Stool	HFMD	OM885401	This study
128	J	USA/2015/CA-RGDS-1050	2015	USA	stool	unknown	MN166093	GenBank
