# Supplementary material for: Molecular Epidemiology and Evolution of Coxsackievirus A9
Source: Viruses. 2022 Apr 15;14(4):822. doi: 10.3390/v14040822 (PMC9024771; doi:10.3390/v14040822)
Supplement: Supplementary file 1 [file viruses-14-00822-s001.zip › viruses-1645085-supl. tables/viruses-1645085-Table S3.pdf]

Table S3. 165 isolates used for phylogeographic analysis of CVA9

No.	Sample ID	Isolation Year	Country of Origin	Sample Type	Clinical Symptoms	GenBank Accession	Source
1	NIV099401	2009	India	Stool	Acute gastroenteritis	KF413000	GenBank
2	CO62	1962	UK	Unknown	N/A	JN996502	GenBank
3	CO79	1979	UK	Unknown	N/A	JN996501	GenBank
4	Cuba47of93	1993	Cuba	CSF	Neuropathy	AY466031	GenBank
5	Cuba23of00	2000	Cuba	CSF	Meningitis	AY466033	GenBank
6	Cuba689of93	1993	Cuba	CSF	Neuropathy	AY466032	GenBank
7	Cuba35of93	1993	Cuba	CSF	Neuropathy	AY466030	GenBank
8	Cuba270of90	1990	Cuba	CSF	Meningitis	AY466024	GenBank
9	Cuba267of90	1990	Cuba	CSF	Meningitis	AY466029	GenBank
10	Cuba163of91	1991	Cuba	CSF	Meningitis	AY466028	GenBank
11	Cuba135of91	1991	Cuba	CSF	Meningitis	AY466027	GenBank
12	Cuba96of91	1991	Cuba	CSF	Meningitis	AY466022	GenBank
13	Cuba45of91	1991	Cuba	CSF	Meningitis	AY466025	GenBank
14	Cuba450of90	1990	Cuba	CSF	Meningitis	AY466023	GenBank
15	Cuba100of91	1991	Cuba	CSF	Meningitis	AY466026	GenBank
16	2-MRS2013	2013	France	CSF	AM	KM201659	GenBank
17	RO-609-4-80	1980	Romania	stool	N/A	LS451285	GenBank
18	CO85	1985	UK	CSF	N/A	JN996500	GenBank
19	CO87	1987	UK	Unknown	N/A	JN996499	GenBank
20	A16-52PD	2010	India	Stool	Persistent diarrhea	KF177114	GenBank
21	NIV56210	2005	India	Stool	AFP	KF412927	GenBank
22	A450D	2011	India	Unknown	Acute diarrhea	JX513563	GenBank
23	263/CSF/CVA9/RUS/Omsk/2012	2012	Russia	CSF	AM	KU133575	GenBank
24	382/CSF/CVA9/RUS/Omsk/2013	2013	Russia	CSF	AM	KU133620	GenBank
25	06.109.3344	2006	Australia	Unknown	AM	FJ868282	GenBank
26	NSW-V20-2008-CVA9	2008	Australia	Stool	N/A	MF678309	GenBank
27	NSW-V56-2006-CVA9	2006	Australia	Stool	N/A	MF678346	GenBank
28	CVA9/EBMSV0003/THA/2010	2010	Thailand	Throat	Respiratory diseases	KU574638	GenBank
29	Patient FJ98-90	2004	FJ/China	Unknown	Unknown	AY573577	GenBank
30	Patient FJ00-127	2004	FJ/China	Unknown	Unknown	AY573578	GenBank
31	97089/SD/CHN/1997/CA9	1997	SD/China	Unknown	N/A	GQ329727	GenBank
32	01332/SD/CHN/2001/CA9	2001	SD/China	Unknown	N/A	GQ329730	GenBank
33	CVA9_CF027040_FRA07	2007	France	Throat	N/A	HF948081	GenBank
34	064/LY/CHN/AM/10/CA9	2010	SD/China	CSF	AM	KF150145	GenBank
35	CVA9/FJZZ122/CHN/2011	2011	FJ/China	Stool	HFMD	MG922505	GenBank
36	YZ047/SD/CHN/2005/CA9	2005	SD/China	Stool	HFMD	GQ246517	GenBank
37	CVA9_Alberta_2013	2013	Canada	CSF	AM	JQ837913	GenBank
38	97186/SD/CHN/1997/CA9	1997	SD/China	Stool	HFMD	GQ329728	GenBank
39	00365/SD/CHN/2000/CA9	2000	SD/China	Stool	HFMD	GQ329729	GenBank
40	04318/SD/CHN/2004/CA9	2004	SD/China	Stool	HFMD	GQ329731	GenBank
41	USA/MI/2005-23029	2005	USA	Unknown	N/A	MH752987	GenBank
42	NSW-V14-2009-CVA9	2009	Australia	Stool	N/A	MF678303	GenBank
43	1-D7-CA9	2008	TW/China	Throat	Rash	KT353721	GenBank
44	61238-7074	2008	TW/China	Throat	Rash	MF422557	GenBank
45	JB14080245	2008	GD/China	Stool	HFMD	KC867074	GenBank
46	Gansu05-1/GS/CHN/2005	2005	GS/China	CSF	AM	GQ294574	GenBank
47	A242/YN/CHN/2009	2009	YN/China	Feces	AM	KM890278	GenBank
48	2249/PL12/3013	2013	Poland	Stool	AFP	KU189253	GenBank
49	CVA9/PMKA0512/THA/2010	2010	Thailand	Throat	Respiratory diseases	KU574637	GenBank

50	CVA9/PMKA0420/THA/2010	2010	Thailand	Throat	Respiratory diseases	KU574636	GenBank
51	CVA9_Alberta_2010	2010	Canada	CSF	AM	JQ837914	GenBank
52	253/JN/CHN/AM/10/CA9	2010	SD/China	CSF	AM	KF246748	GenBank
53	A14/YN/CHN/2010	2010	YN/China	CSF	AM	MF098518	GenBank
54	A73/YN/CHN/2010	2010	YN/China	CSF	AM	MF098523	GenBank
55	NSW-V41-2013-CVA9	2013	Australia	Stool	N/A	MF678330	GenBank
56	P73/ZS/CHN/2011	2011	China/GX	Stool	HFMD	MN018193	GenBank
57	024/LS/CHN/AM/08/CA9	2008	SD/China	CSF	AM	KF246747	GenBank
58	USA/2019-23312	2019	USA	Liver	Unknown	MN896915	GenBank
59	USA/2019-23313	2019	USA	Liver	Unknown	MN896916	GenBank
60	USA/2019-23314	2019	USA	Liver	Unknown	MN896917	GenBank
61	CLI-B1-10-CV-A9	2017	UK	CSF	AM	MT641360	GenBank
62	16C2	2016	USA	Unknown	N/A	KY674974	GenBank
63	16C8	2016	USA	Unknown	N/A	KY674976	GenBank
64	CVA9/FJQZ126/CHN/2011	2011	FJ/China	Stool	HFMD	MG922506	GenBank
65	A108/YN/CHN/2009	2009	YN/China	Feces	AM	KM890277	GenBank
66	A744/YN/CHN/2009	2009	YN/China	CSF	AM	MN686207	GenBank
67	CVA9/FJZZ056/CHN/2011	2011	FJ/China	Stool	HFMD	MG922504	GenBank
68	CV-A9/P351/2013/China	2013	ZJ/China	CSF	AM	KP289437	GenBank
69	LHY03	2010	JS/China	Unknown	HFMD	KP266574	GenBank
70	JB141230009	2012	GD/China	Stool	HFMD	KC867075	GenBank
71	071/LY/CHN/AM/10/CVA9	2010	SD/China	CSF	AM	KF150146	GenBank
72	C1/LC/2019	2019	SD/China	CSF	AM	MT950542	GenBank
73	C23/LC/CHN/2019	2019	SD/China	CSF	AM	MT950564	GenBank
74	C39/LC/CHN/2018	2018	SD/China	CSF	AM	MT646125	GenBank
75	ZG2018-176-CVA9	2018	SC/China	Stool	HFMD	MW179455	GenBank
76	JB141230166	2012	GD/China	Stool	HFMD	KC867076	GenBank
77	JB141230172	2012	GD/China	Stool	HFMD	KC867077	GenBank
78	JB141230177	2012	GD/China	Stool	HFMD	KC867078	GenBank
79	CVA9/P56/2013/China	2013	ZJ/China	Stool	HFMD	KP289434	GenBank
80	CVA9/FJZ463/CHN/2020	2014	FJ/China	Stool	HFMD	MG922507	GenBank
81	DY2016-50-CVA9	2014/2019	SC/China	Stool	N/A	MW179442	GenBank
82	CV-A9/P220/2013/China	2013	ZJ/China	Stool	HFMD	KP290111	GenBank
83	A34-YN-CHN-2014	2014	YN/China	Stool	HFMD	LC013414	GenBank
84	A34-YN-CHN-2014	2014	YN/China	Stool	HFMD	LC411969	GenBank
85	120-YN-CHN-2014JK	2014	YN/China	Homo	Healthy children	LC120871	GenBank
86	CVA9/FJQZ182/CHN/2012	2012	FJ/China	Stool	HFMD	MG922503	GenBank
87	C28/LC/CHN/2019	2019	SD/China	CSF	AM	MT950569	GenBank
88	C38/LC/CHN/2019	2019	SD/China	CSF	AM	MT950579	GenBank
89	C18-YN-CHN-2018	2018	YN/China	Stool	HFMD	LC481444	GenBank
90	C20-YN-CHN-2018	2018	YN/China	Stool	HFMD	LC481445	GenBank
91	DY2016-18-CVA9	2016	SC/China	Homo	Healthy children	MW179429	GenBank
92	DY2016-21-CVA9	2014/2019	SC/China	Unknown	Healthy child	MW179431	GenBank
93	A280-KM-CHN-CVA9	2018	YN/China	Unknown	HFMD	LC516768	GenBank
94	C4/LC/CHN/2019	2019	SD/China	CSF	AM	MT950545	GenBank
95	C16/LC/CHN/2019	2019	SD/China	CSF	AM	MT950557	GenBank
96	SF149	2016	SC/China	Stool	Healthy children	LC361281	GenBank
97	ZJ154	2016	ZJ/China	Stool	N/A	LC361282	GenBank
98	ZJ160	2016	ZJ/China	Stool	N/A	LC361283	GenBank
99	China/GS/2015/05	2015	GS/China	Stool	HFMD	OM885385	This study
100	China/GS/2015/61	2015	GS/China	Stool	HFMD	OM885386	This study
101	China/GS/2015/254	2015	GS/China	Stool	HFMD	OM885387	This study
102	China/GX/2019/175	2019	GX/China	Stool	HFMD	OM885388	This study

103	China/HeB/2019/98	2019	HeB/China	Stool	HFMD	OM885389	This study
104	China/SaX/2019/49	2019	SaX/China	Stool	HFMD	OM885390	This study
105	China/SD/2015/41	2015	SD/China	Stool	HFMD	OM885392	This study
106	China/SD/2016/246	2016	SD/China	Stool	HFMD	OM885391	This study
107	China/SD/2019/074	2019	SD/China	Stool	HFMD	OM885393	This study
108	China/SD/2019/114	2019	SD/China	Stool	HFMD	OM885394	This study
109	China/SD/2019/130	2019	SD/China	Stool	HFMD	OM885395	This study
110	China/SD/2019/323	2019	SD/China	Stool	HFMD	OM885396	This study
111	China/SD/2019/403	2019	SD/China	Stool	HFMD	OM885397	This study
112	China/SD/2019/437	2019	SD/China	Stool	HFMD	OM885398	This study
113	China/XJ/2015/83	2015	XJ/China	Stool	HFMD	OM885399	This study
114	China/XJ/2017/117	2015	XJ/China	Stool	HFMD	OM885400	This study
115	China/XJ/2018/101	2015	XJ/China	Stool	HFMD	OM885402	This study
116	China/YN/2014/131	2014	YN/China	Stool	HFMD	OM885403	This study
117	99.358.0740	1999	Australia	Unknown	N/A	GU142874	GenBank
118	CF315290013_FRA15-10-16_CV-A9	2015	France	CSF	Meningitis	MK086198	GenBank
119	408/CSF/CVA9/RUS/Omsk/2013	2013	Russia	CSF	AM	KU133635	GenBank
120	SPb_5005/13EVI/RKa-32/14/RU	2013	Russia	CSF	AM	KU841453	GenBank
121	297/CSF/CVA9/RUS/Omsk/2012	2012	Russia	CSF	AM	KU133591	GenBank
122	302/CSF/CVA9/RUS/Omsk/2012	2012	Russia	CSF	AM	KU133594	GenBank
123	299/CSF/CVA9/RUS/Omsk/2012	2012	Russia	CSF	AM	KU133593	GenBank
124	309/CSF/CVA9/RUS/Omsk/2012	2012	Russia	CSF	AM	KU133597	GenBank
125	270/CSF/CVA9/RUS/Omsk/2012	2012	Russia	CSF	AM	KU133580	GenBank
126	325/CSF/CVA9/RUS/Omsk/2012	2012	Russia	CSF	AM	KU133606	GenBank
127	China/XJ/2018/99	2018	XJ/China	Stool	HFMD	OM885401	This study
128	USA/2015/CA-RGDS-1050	2015	USA	Stool	Unknown	MN166093	GenBank
129	Monastir/F26/CSF_5.05_TUN_2011	2011	Tunisia	CSF	AM	LN713452	GenBank
130	Monastir/F26/CSF_4.13_TUN_2011	2011	Tunisia	CSF	AM	LN713453	GenBank
131	CF152050_TH_FRA10	2010	France	Throat	AM	LN876173	GenBank
132	CF035013_F_FRA11	2011	France	Stool	AM	LN876174	GenBank
133	CF193068_PL_FRA11	2011	France	Serum	AM	LN876175	GenBank
134	CF215004_CSF_FRA11	2011	France	CSF	AM	LN876176	GenBank
135	CF185079_TH_FRA07	2007	France	Throat	AM	LN876177	GenBank
136	409-CSF/CVA9/RUS/Omsk/2013	2013	Russia	CSF	AM	KU133636	GenBank
137	393/CSF/CVA9/RUS/Omsk/2013	2013	Russia	CSF	AM	KU133625	GenBank
138	313/CSF/CVA9/RUS/Omsk/2012	2012	Russia	CSF	AM	KU133600	GenBank
139	312/CSF/CVA9/RUS/Omsk/2012	2012	Russia	CSF	AM	KU133599	GenBank
140	296/CSF/CVA9/RUS/Omsk/2012	2012	Russia	CSF	AM	KU133590	GenBank
141	261/CSF/CVA9/RUS/Omsk/2012	2012	Russia	CSF	AM	KU133574	GenBank
142	03-132NPR2	2003	Japan	Unknown	Unknown	AB167978	GenBank
143	03-144NPC3	2003	Japan	Unknown	Unknown	AB167979	GenBank
144	03-171FCR2	2003	Japan	Unknown	Unknown	AB167980	GenBank
145	148–00	2000	China/YN	Stool	AFP	AB268121	GenBank
146	49–98	1998	China/YN	Stool	AFP	AB268123	GenBank
147	78–97	1997	China/YN	Stool	AFP	AB268124	GenBank
148	CA9/25-0066/Osaka/2013	2013	Japan	Throat	Unknown	AB908274	GenBank
149	TR081056-05	2005	France	Unknown	Unknown	AM236967	GenBank
150	CF186011/CSF_4.96_FRA08	2008	France	CSF	AM	HG793656	GenBank
151	CF239034/CSF_4.96_FRA08	2008	France	CSF	AM	HG793657	GenBank
152	CF179026/CSF_6.13_FRA10	2010	France	CSF	AM	HG793658	GenBank
153	CF183009/CSF_5.54_FRA10	2010	France	CSF	AM	HG793659	GenBank
154	CF242122/CSF_4.10_FRA10	2010	France	CSF	AM	HG793660	GenBank
155	CF192070/CSF_6.21_FRA11	2011	France	CSF	AM	HG793661	GenBank
156	CF193055/CSF_5.29_FRA11	2011	France	CSF	AM	HG793662	GenBank

157	CF197008/CSF_5.28_FRA11	2011	France	CSF	AM	HG793663	GenBank
158	CF207004/CSF_5.64_FRA11	2011	France	CSF	AM	HG793664	GenBank
159	N-451A	2007–09	India	Unknown	Unknown	JN203525	GenBank
160	N-452	2007–09	India	Unknown	Unknown	JN203526	GenBank
161	N-656	2007–09	India	Unknown	Unknown	JN203527	GenBank
162	N-683	2007–09	India	Unknown	Unknown	JN203528	GenBank
163	N-828	2007–09	India	Unknown	Unknown	JN203529	GenBank
164	N-924	2007–09	India	Unknown	Unknown	JN203530	GenBank
165	A16-53PD	2010	India	Stool	Diarrhea	KF177115	GenBank
