# Supplementary material for: Molecular Epidemiology and Evolution of Coxsackievirus A9
Source: Viruses. 2022 Apr 15;14(4):822. doi: 10.3390/v14040822 (PMC9024771; doi:10.3390/v14040822)
Supplement: Supplementary file 1 [file viruses-14-00822-s001.zip › viruses-1645085-supl. tables/viruses-1645085-Table S4.pdf]

Table S4. The results of the Bayes test did by SPREAD

FROM	TO	BAYES_FACTOR(BF)	POSTERIOR PROBABILITY
**USA**	Thailand	2977.8214009700323	0.9958893456282635
**USA**	UK	2621.8556008631185	0.995333851794245
**France**	Romania	1320.650495412015	0.9907788023552938
**USA**	Tunisia	1116.628788982404	0.9891123208532385
**USA**	China	1083.0965085197197	0.9887790245528275
**Tunisia**	Japan	654.179587741939	0.9815576047105877
**USA**	France	549.303455062889	0.9781135429396733
**India**	USA	468.72681343374126	0.9744472836351517
**USA**	Poland	118.79182067995194	0.906232640817687
**Japan**	USA	18.31287666196111	0.5983779580046662
**Japan**	Canada	17.537251296776425	0.5879346739251194
**Poland**	Russia	12.321398797824637	0.5006110432174203
**Japan**	India	11.650288094627882	0.48661259860015554
**Canada**	India	7.667938717576347	0.3841795356071548
**France**	India	6.221760840468351	0.33607376958115764
**USA**	Russia	5.458426690374711	0.3075213865126097
**France**	Tunisia	5.294782547333201	0.3010776580379958
**India**	Canada	4.527495923669128	0.26919231196533716
**India**	Cuba	4.1329928202566215	0.2516387068103544
**Japan**	Cuba	4.079525096009087	0.24919453394067326
**India**	Australia	4.077103015076382	0.24908343517386958
**France**	Australia	4.0480938472549886	0.2477502499722253
**India**	France	3.719413888531312	0.23230752138651262
**China**	Poland	3.5158197594151237	0.22241973114098434
**Cuba**	Australia	3.4103790036224284	0.21719808910121097
**Japan**	France	3.308534932716999	0.2120875458282413
**France**	Cuba	3.2123474868260335	0.20719920008887902
